# Trends and characteristics of oral and maxillofacial injuries in Nigeria: a review of the literature

**DOI:** 10.1186/1746-160X-1-7

**Published:** 2005-10-04

**Authors:** Wasiu Lanre Adeyemo, Akinola Ladipo Ladeinde, Mobolanle Olugbemiga Ogunlewe, Olutayo James

**Affiliations:** 1 Department of Oral and Maxillofacial Surgery, Lagos University Teaching Hospital, P.M.B. 12003, Lagos, Nigeria; 2 Department of Oral and Maxillofacial Surgery, College of Medicine, University of Lagos, P.M.B. 12003, Lagos, Nigeria

## Abstract

**Background:**

The etiology of maxillofacial injuries varies from one country to another and even within the same country depending on the prevailing socioeconomic, cultural and environmental factors. Periodic verification of the etiology of maxillofacial injuries helps to recommend ways in which maxillofacial injuries can be averted. The aim of the present study is therefore to analyse the characteristics and trends of maxillofacial injuries in Nigeria based on a systematic review of the literature.

**Methods:**

A literature search using MEDLINE was conducted for publications on maxillofacial injuries in Nigeria. The relevant references in these publications were manually searched for additional non-Medline articles or abstracts. Forty-two studies met the inclusion criteria and the full-texts of these articles were thoroughly examined. Due to lack of uniformity and consistency in assessment and measurement variables, and treatment modalities in most of the studies, it was impossible to apply the traditional methods of a systematic review. Therefore, a narrative approach was conducted to report the findings of the included studies.

**Results:**

Although, other causes like assaults, sport injuries, and industrial accidents increased in numbers, throughout the period between 1965 and 2003, road traffic crashes remained the major etiological factor of maxillofacial injuries in all regions, except northeastern region where assault was the major cause. A significant increase in motorcycles related maxillofacial injuries was observed in most urban and suburban centres of the country. Animal attacks were not an unusual cause of maxillofacial injuries in most parts of northern Nigeria. Patients in the age group of 21–30 years were mostly involved. A strong tendency toward an equal male-to-female ratio was observed between earlier and later periods.

**Conclusion:**

Road traffic crashes remain the major cause of maxillofacial injuries in Nigeria, unlike in most developed countries where assaults/interpersonal violence has replaced road traffic crashes as the major cause of the injuries. There is a need to reinforce legislation aimed to prevent road traffic crashes and the total enforcement of existing laws to reduce maxillofacial injuries among children and adults. Special attention should also be paid by the authority to improve the socioeconomic conditions of Nigerian populace.

## Background

Skeletal and soft tissue injuries of the face constitute quite a significant portion of the workload of the oral and maxillofacial surgeons in Nigeria [1]. Being the most exposed part of the body, the face is particularly vulnerable to such injuries, 20–60% of all those involved in automobile accidents having some level of facial fractures [2,3]. Surveys of facial injuries have shown that the etiology varies from one country to another and even within the same country depending on the prevailing socioeconomic, cultural and environmental factors [4-6]. Earlier studies from Europe and America revealed that road traffic crashes (RTC) were the most frequent cause of facial injuries [7,8]. However, more recent studies have shown that assault is now the most common cause of maxillofacial injuries in developed countries [9-11], whereas traffic accidents remain the most frequent cause in many developing countries [12-19].

Periodic verification of the etiology of maxillofacial injuries helps to assess the proficiency of road safety measures such as speed limit, drunk driving, and seat beat belt laws and the behavioural patterns of the people in different countries and helps to recommend other ways in which injuries to the face can be averted [20].

The aim of the present study is therefore to analyse the characteristics and trends of maxillofacial injuries in Nigeria based on a systematic review of the literature.

## Methods

A computerized literature search using MEDLINE was conducted for publications on maxillofacial injuries in Nigeria published between 1970 and 2005. For this search, the medical subject headings "maxillofacial injuries" or "maxillofacial fractures" or "mandible fractures" or "middle-third fractures" or "facial fractures" or "zygoma fractures" were combined with "Nigeria" or "Africa". The Boolean operator 'AND' was used to combine and narrow the searches. We manually searched the references in these articles to look for additional relevant non-Medline articles or abstracts. The full-texts of all these articles were thoroughly examined. Personal contacts were also made with institutions and investigators of previous studies for missing data and also for the provision of articles found suitable for the review, but not readily available to us. One author (WLA) conducted the literature search. All the authors agreed upon inclusion and exclusion criteria.

### Inclusion criteria

1. Availability of the full-text article

2. Retrospective or prospective studies

3. All age groups (Children and adults)

4. Civilian-type injuries

Publications on maxillofacial injuries sustained during Nigerian civil war were excluded from the review.

### Assessment of the studies

**Figure 1:**
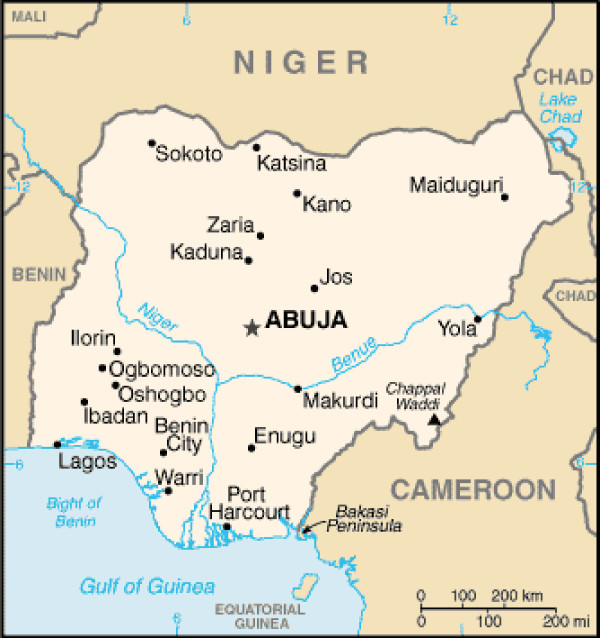
Map of Nigeria (source: CIA's The World Factbook). Ife (not shown) lies in the north east of Ibadan below Oshogbo.

A total of 44 full-text articles and abstracts were identified. Two articles on maxillofacial injuries sustained during the Nigerian civil war were excluded. A total of 42 publications published between January 1977 and April 2005, which satisfied the inclusion criteria were, therefore included in the review. These included 34 Medline and 8 non-Medline articles. These publications were based on patients seen and treated between 1965 and 2003 from different centers of the six geopolitical zones of the country (Figure 1) including: Ibadan, south west (SW) [18,19,21-35], Lagos (SW) [36-40], Ife (SW) [1,41-46], Kaduna, north central (NC) [47-50], Sokoto, north west (NW) [51,52], Maiduguri, north east (NE) [4,53,54], Enugu, south east (SE) [15,16,55], and Benin city, south south (SS) [56] (Table 1).

**Table 1:** Location of investigations, aetiology of injury and gender distribution.

Author (Ref.-No.)	Location^a^	Major cause of injury	2^nd ^major cause of injury	% motorcycle related	Male/female ratio (%)	Tissue affected
Ajagbe et al. (21)	Ibadan (SW)	RTC (63)	Falls (19)	10	2.1:1	Hard Tissue
Nwoku et al. (36)	Lagos (SW)	RTC	##	###	3:1	Hard/Soft Tissues
Ajagbe et al. (34)	Ibadan (SW)	RTC	Falls	15.7	3:1	Hard Tissue
Adekeye (47)	Kaduna (NC)	RTC (76)	Assaults (13)	22	16.9:1	Hard/Soft Tissues
Adekeye (48)	Kaduna (NC)	RTC (82)	Falls	###	24:1	Hard Tissue
Adekeye (49)	Kaduna (NC)	RTC	##	###	2:1	Hard Tissue
Nyako (23)	Ibadan (SW)	RTC (77)	Assaults (9)	10.6	6.4:1	Hard Tissue
Odusanya (41)	Ife (SW)	RTC (53)	Falls	22.1	5.4:1	Hard Tissue
Abiose (22)	Ibadan (SW)	RTC (81)	Assaults (9)	###	5.5:1	Hard Tissue
Akinwande (37)	Lagos (SW)	RTC (65)	Assaults (12)	18.5	4.2:1	Hard/Soft Tissues
Abiose (32)	Ibadan (SW)	RTC (81)	Assaults (7)	###	14:1	Hard Tissue
Arotiba (38)	Lagos (SW)	RTC (100)	#	6.3	2.3:1	Hard Tissue
Arotiba (39)	Lagos (SW)	RTC (63)	Assaults (20)	4	2.1:1	Hard Tissue
Oji (15)	Enugu (SE)	RTC (83)	Assaults (8)	21	3:1	Hard Tissue
Ogunbodede (52)	Sokoto (NW)	^b^Camel bite	#	#	#	Hard/Soft Tissues
Denloye et al. (33)	Ibadan (SW)	RTC (47)	Falls (41)	###	1.8:1	Hard/Soft Tissues
Ugboko et al. (1)	Ife (SW)	RTC (72)	Falls (11)	14.5	4.1:1	Hard/Soft Tissues
Akinwande et al. (40)	Lagos (SW)	^b^Gunshots (100)	#	#	5.1:1	Hard/Soft Tissues
Oji (55)	Enugu (SE)	RTC (28)	Assaults (25)	5	2.6:1	Hard Tissue
Ugboko et al. (46)	Ife (SW)	RTC (50)	Falls (31)	1.9	6.4:1	Hard/Soft Tissues
Oji (16)	Enugu (SE)	RTC (83)	Assaults (8)	21	3:1	Hard Tissue
Olasoji (53)	Maiduguri (NE)	^b^Assaults (100)	#	#	2.5:1	Hard/Soft Tissues
Ugboko et al. (42)	Ife (SW)	^b^Gunshots (100)	#	#	21:1	Hard /Soft Tissues
Fasola et al. (28)	Ibadan (SW)	^b^Sports (100)	#	#	4.1:1	Hard Tissue
Fasola et al. (30)	Ibadan (SW)	RTC (38)	Falls (25)	###	2.6:1	Soft Tissue
Fasola et al. (19)	Ibadan (SW)	RTC (79)	Assaults (9)	###	7.6:1	Hard/Soft Tissues
Fasola et al. (25)	Ibadan (SW)	RTC (52)	Falls (24)	3.2	2.6:1	Hard/Soft Tissues
Fasola et al. (26)	Ibadan (SW)	RTC (53)	Falls (24)	###	2.8:1	Hard Tissue
Olasoji et al. (4)	Maiduguri (NE)	Assaults (48)	RTC (36)	9	2.2:1	Hard /Soft Tissues
Olasoji et al. (54)	Maiduguri (NE)	RTC (54)	Falls (25)	2	7.5:1	Hard/Soft Tissues
Ugboko et al. (51)	North (NE, NW, NC)	^b^Animal attacks	#	#	4:1	Hard/Soft Tissues
Oginni et al. (43)	Ife (SW)	^b^Dog bites	#	#	5:3	Soft Tissue
Oginni et al. (44)	Ife (SW)	Falls (38)	RTC (33)	#	1.4:1	Soft Tissue
Fasola et al. (29)	Ibadan (SW)	RTC (76)	Assaults (9)	###	2.7:1	Hard/Soft Tissues
Fasola et al. (31)	Ibadan (SW)	RTC (82)	Sports (8)	###	5.3:1	Hard Tissue
Fasola et al. (18)	Ibadan (SW)	RTC (69)	Assaults (12)	20.6	3.3:1	Hard Tissue
Fasola et al. (24)	Ibadan (SW)	RTC (69)	#	15.1	2.9:1	Hard Tissue
Fasola et al. (27)	Ibadan (SW)	RTC (59)	Falls (21)	1.9	1:1	Hard Tissue
Saheeb et al. (56)	Benin (SS)	RTC (66)	Assaults (10)	26.5	2.7:1	Hard /Soft Tissues
Adebayo et al. (50)	Kaduna (NC)	RTC (56)	Falls (24)	###	4.7:1	Hard Tissue
Bankole et al. (35)	Ibadan (SW)	Falls (66)	RTC (18)	###	2.3:1	Soft Tissue
Ugboko et al. (45)	Ife (SW)	RTC (74)	Falls/Assaults (14)	9.4	6:1	Hard Tissue

^a^(SW) South-west, (SE) South-east (SS) South-south, (NW) North-west, (NE) North-east, (NC) North-central

^b^Publications on a single specific etiology

RTC = road traffic crash

# = not applicable

## = not specified

### = not separately classified

A protocol was prepared to identify the following features of each study: type of participants (i.e. adults or children or both groups), number of injuries analyzed, etiology of injury, peak age of incidence, gender predilection, site of injury, target population, as well as period and location of the study (Table 1, 2). Treatment modalities were also assessed.

**Table 2:** Type of included study, number of patients analyzed, target population and peak age of incidence.

Author (Ref.-No.)	Type of study	n of patients	Target population	Bone mostly affected (%)	Peak age of incidence, years (%)
Ajagbe et al (21)	retrospective	203	total	mandible (60.5)	21–30 (32)
Nwoku et al (36)	retrospective	84	total	mandible (90)	##
Ajagbe et al (34)	retrospective	324	total	mandible (60)	21–30
Adekeye (47)	prospective	1447	total	mandible (62.5)	21–30 (56)
Adekeye (48)	retrospective	337	total	#	21–40 (80)
Adekeye (49)	retrospective	85	Children	mandible	>10
Nyako (23)	retrospective	341	total	mandible (73)	21–30 (46)
Odusanya (41)	retrospective	231	total	mandible (67)	21–30
Abiose (22)	retrospective	104	total	mandible (75)	21–30 (43)
Akinwande (37)	prospective	208	total	mandible	21–30 (51)
Abiose (32)	retrospective	59	total	#	21–30
Arotiba (38)	prospective	128	total	mandible (62)	20–29 (>40)
Arotiba (39)	prospective	202	total	mandible (64)	20–29 (40)
Oji (15)	retrospective	900	total	mandible (42)	21–30 (36)
Ogunbodede (52)	case report	1	#	#	#
Denloye et al (33)	retrospective	106	Children	mandible	0–8 (62)
Ugboko et al (1)	retrospective	442	total	mandible (64)	21–30 (39)
Akinwande et al (40)	prospective	35	total	mandible	20–34 (66)
Oji (55)	retrospective	40	Children	mandible (89)	9–11 (40)
Ugboko et al (46)	retrospective	52	Children	mandible (62)	12–14 (50)
Oji (16)	retrospective	900	total	mandible (53)	21–30 (36)
Olasoji (53)	retrospective	105	total	mandible (43)	20–29 (42)
Ugboko et al (42)	retrospective	22	total	Zygoma (27)	21–40
Fasola et al (28)	retrospective	77	total	mandible (54.4)	21–30 (52)
Fasola et al (30)	retrospective	831	total	#	21–30 (33)
Fasola et al (19)	prospective	103	total	#	21–30 (47)
Fasola et al (25)	retrospective	93	children	mandible (86)	11–16 (54)
Fasola et al (26)	retrospective	72	children	#	12–16 (57)
Olasoji et al (4)	prospective	306	total	mandible (66)	21–30 (41)
Olasoji et al (54)	retrospective	102	Children	mandible (73)	12–15 (54)
Ugboko et al (51)	retrospective	34	total	mandible (56)	11–30 (74)
Oginni et al (43)	retrospective	8	children	#	##
Oginni et al (44)	retrospective	174	children	#	##
Fasola et al (29)	^a^retrospective	531	total	#	21–30 (39)
Fasola et al (31)	prospective	76	total	#	21–30 (51)
Fasola et al (18)	^b^pro/retrospective	824	total	mandible (75)	21–30 (36)
Fasola et al (24)	prospective	159	total	mandible	21–30 (36)
Fasola et al (27)	retrospective	53	adults	mandible (96)	60–70 (77)
Saheeb et al (56)	retrospective	250	total	mandible (65)	20–30 (32)
Adebayo et al (50)	retrospective	443	total	mandible (64)	20–39 (65)
Bankole et al (35)	retrospective	64	children	#	0–5
Ugboko et al (45)	retrospective	128	total	#	21–30 (38)

^a ^analysis of concomitant injuries in patients with maxillofacial fractures

^b^comparative study

total = all age groups

# = not applicable

## = not specified

Most of the studies lack uniformity and consistency in assessment and measurement variables (information bias) and treatment modalities. The age bracket of patients considered as "children" by several investigators varied considerably (Fasola et al [26], 16 years and below; Oji [55], under 11 years; Olasoji, under 15 years; Ugboko et al, 14 years and below [46]; Denloye et al [33], less than 17 years; Oginni et al [44], 15 years and below). Repetition of the same data in different studies was also observed. While most of the published articles focused only on hard tissue injuries, few others reported on either hard and soft tissue injuries or soft tissue only (Table 1). Although, the majority of the patients in the studies were treated by closed reduction and fixation methods, uniformity in treatment was lacking. Due to the heterogeneity of the study methodologies in this review it was not possible to apply the traditional methods of a systematic review. A meta-analysis is only suitable if there is sufficient similarity in the populations studied and the measurements used. This was not the case with the studies identified in this review. Therefore, a narrative approach was taken to report the findings of the included studies.

Data was analysed using the SPSS for window (version 11.5; SPSS Inc, Chicago, IL) statistical software package. Descriptive statistics and the non-parametric chi square test were used to analyse the incidence of injuries in different time periods. The critical level of significance was set at p < .05.

## Results

Of the 42 articles reviewed, 31 were retrospective studies, 9 prospective, 1 article was a case report and 1 article was a comparative study of a prospective and a retrospective data. Road traffic crash (RTC) was the major cause of maxillofacial injuries in both children and adults in all the zones of the country with the exception of north eastern states where assault was the major cause of injuries (Table 1). Although, motor vehicles were responsible for most cases of RTC, motorcycle related injuries increased significantly between 1965 and 2003. Between 1965 and 1999 in Ibadan, the number of motorcycle-related maxillofacial injuries increased by a factor of 2.6, and more significant cases (p = .02) of motorcycle related injuries were recorded in 1978–1982 period compared to 1995–1999 (Table 3). In Enugu (SE) Nigeria, between 1985 and 1995, the number of motorcycle related maxillofacial injuries increased by a factor of 1.6 (Table 3). An increase in the number of motorcycle related maxillofacial injuries was also observed between 1973 and 2000, and between 1976 and 1995 in Kaduna (NC) [48,51] and Ife (SW) [1,41] respectively. In Benin (SS) [56] and Lagos (SW) [37], 26.5% and 19.0% of cases with maxillofacial injuries were involved in motorcycle related crashes respectively, and motorcycle passengers sustained more severe injuries than other vehicle users [37,56].

**Table 3:** Analysis of road traffic injuries due to motor vehicles and motorcycles between 1965 and 1999 in Ibadan^a ^and between 1985 and 1995 in Enugu^b^.

	**IBADAN (South-west, Nigeria)**			
Types of automobile involved	Study period			

	1965–1975	1978–1982	1982–1984	1995–1999

Motor vehicles	46.3%	84.9%	80%	63.4%
Motorcycles	7.8%	10.6%	#	20.6%*
				
	**ENUGU (South-east, Nigeria)**			

	Study period			

	1985–1990		1991–1995	

Motor vehicles	59%		59%	
Motorcycles	16%		25%	

^a ^adapted from Abiose [22], Ajagbe et al [21], Fasola et al [18] and Nyako [23]

^b ^adapted from Oji [16]

# = not specified

* statistically significant (p = 0.02)

Pedestrian related maxillofacial fractures also increased in major cities across the country. In Ibadan (SW), an increase by a factor of 3.2 was reported between 1978 and 1999 [18,23] and in Lagos (SW), 35.6% (1983–1986) and 28.1% (1989–1992) of patients involved in RTC were pedestrians hit by vehicles [37,38].

Assaults were the second most common cause of injuries in most centres followed by falls (Table 1). Falls were important causes of injuries in children. Increase in the number of patients who sustained injuries as a result of assaults, falls, sports injuries and industrial accidents was observed in most centers over the years [1,18,35,37,46,48,51,53]. Animal attacks were also a frequent cause of maxillofacial injuries especially in northern part of the country [4,43,45,52].

The peak age of incidence of maxillofacial injuries was 21–30 years in most centers followed by 31–40 years. In children, injuries occurred mostly in children aged > 10 years. More males were affected than females in all age groups. A tendency towards an equal male-to-female ratio was observed between earlier and later studies in most urban centers. A significant reduction in male-to-female ratio from 16.9:1 (1973–1978) to 3:1 (1991–2000) was reported from Kaduna (NC) (Table 1). Another significant reduction in male-to-female ratio from 6.4:1 (1978–1982) to 3.3:1 (1995–1999) was reported from Ibadan (SW) (Table 1).

The Mandible was the most frequently involved bone in maxillofacial fractures in all the centers across the country, and the most frequently involved middle-third bone was the zygoma [1,18,22,32,50]. The LeFort I fracture was the most common of the LeFort fracture types [1,4,22]. Analysis of fracture of the mandible revealed mandibular body as the most frequently involved part, followed by symphyseal/ parasymphyseal region [1,4,18,23,25,37-39,46,47,50]. Dentoalveolar and condylar fractures were less frequently reported. Another remarkable feature of maxillofacial injuries in most reports was extensive soft tissue injuries [30,18,37,38,56].

Closed reduction and dental wiring with arch bars, direct wires and eyelet wires combine with intermaxillary fixation were the most common form of treatment [1,21,34,47,50] for mandibular fractures. Wire osteosynthesis is employed for open reduction and internal fixation of mandibular fractures in few cases [1,21,22,50]. Fractures of the maxillae/LeFort fractures were reduced and fixed by eyelets/arch bars combined with suspension wires and intermaxillary fixation [1,32,34,47,50]. Zygomatic complex fractures were treated either conservatively or by either closed or open reduction with Gillies' temporal approach, lateral coronoid approach or transosseous wiring [21,42,45,47].

## Discussion

The large variations in assessment and measurement variables, as well repetition of data employed by previous investigators of maxillofacial injuries in Nigeria made a systematic review impossible. However, analysis of the previous studies on maxillofacial injuries in Nigeria showed a noticeable trend and characteristic.

Although, road traffic crashes remained the major etiological factor of maxillofacial injuries other causes like assaults, sport injuries and industrial accidents have increased in numbers between 1965 and 2003 in Nigeria. This finding is in agreement with reports from other developing countries where RTC remains the major etiologic factor of maxillofacial injuries [12,13,17], but contrasts reports from developed countries where assaults and interpersonal violence has replaced RTC as the major cause of maxillofacial injuries [6,10,11,18]. Civilian-type maxillofacial injuries were rare prior to Nigerian independence in 1960 [21]. Immediate post independence period witnessed a significant increase in the numbers of motor vehicles imported into the country. It is worthwhile to note that the period from 1965 up to the present time has witnessed a steady increase in the number of second-hand vehicles into Nigeria. Also, lack of enforcement of reshipment inspection rules and regulations has encouraged the importation of vehicles whose road worthiness leaves much to be desired [1]. In addition, the roads are badly maintained, and there is general lack of enforcement of traffic rules and regulations, especially the use of seat belts. Non-usage of protective elements was also thought to be responsible for extensive soft tissue injuries seen in maxillofacial injured patients [18,37,38,56].

Over the last 40 years, there has been a significant increase in the number of maxillofacial injuries that resulted from motorcycle accidents in Nigeria (Table 3). These findings contrast that of others [57] who reported a decrease in the number of motorcyclists involved in maxillofacial injuries. However, Konto et al [58] reported that bicycle related maxillofacial fractures increased by 19.3% between 1981 and 1997 in Finland. The increase in the present study is due to a significant increase in the number of motorcycles plying Nigeria roads. Even in Abuja, the nation's capital, anecdotal evidence has shown that motorcyclists and their passengers are involved in more than 55% of cases of road traffic crashes. In the United States of America (USA), the number of registered motorcycles increased from 600,000 units in 1961 to 3.3 million units in 1971; a 450% increase within a decade [59,60]. This pattern was also recognised in Nigeria when the number increased from 144,480 units to 284,124 units between 1976 and 1981, an increase of almost 200% within 5 years [61]. Motorcycles have become a prominent mode of transportation in both urban and suburban cities in Nigeria. Frequent traffic jams as a result of poor road network in the country have made motorcycles attractive to commuters because motorcycles can pass through narrow ways [18]. However, most of the motorcyclists are unlicensed and often do not follow traffic rules and regulation. Fasola et al [24] reported that only one (3.8%) of the motorcyclists who sustained maxillofacial injuries within Ibadan city (SW) wore a crash helmet while Saheeb and Etetafia [56] reported that none of the motorcyclists and their passengers involved in RTC in Benin city (SS) wore protective helmet.

The number of pedestrians involved in maxillofacial injuries has also been on the increase especially in urban centres unlike reported elsewhere [57]. This is peculiar to the overpopulated cities with few subways and overhead bridges. Therefore, it is relatively common for pedestrians to have to run in oncoming vehicular traffic [18,38]. Konto et al [58] also reported an increase in pedestrian related maxillofacial fractures in their study.

While RTC have been steadily falling in the developed countries, they continue to rise with horrifying speed in the low and middle-income (LMIC) countries of Africa and Asia [62]. The World Health Organisation (WHO) has estimated that nearly 25% of all injury fatalities worldwide are a result of road traffic crashes, with 90% of the fatalities occurring in LMIC [62]. The reductions in RTC in developed countries are largely attributed to a wide range of road safety measures such as seat belt use, traffic calming measures and traffic law enforcement. Therefore, there is an urgent need to get down to what the developed nations have done to reduce/prevent road traffic crashes.

Assaults and falls were the second most common cause of maxillofacial injuries in adults and children respectively in all centres except the north eastern part of the country, where assaults remained a major cause (Table II). Other common causes were sport injuries, industrial accidents and animal attacks. Fasola et al [18] in Ibadan (SW) reported an increase in number of maxillofacial injuries due to assaults, falls, sporting injuries and industrial accidents between 1978 and 1999 by a factor of 1.4, 1.5, 3.5, and 1.5 respectively. Increase in number of assaults related maxillofacial injuries could be attributed to the poor socioeconomic conditions of the country leading to stress and propensity to crime. In fact, the employment rate among college and university graduates has increased from 4% during the early 1970s to 45% currently [4]. Furthermore, the poor capital income of an average Nigerian has decreased by 75% during the past 20–25 years [4,63]. The prevalence of assaults related injury in north eastern Nigeria could be attributed to nomadic form of life style in this region, where animals are moved over several kilometres of land grazing without strict laws guiding their movement thereby destroying cash crops [53]. This frequently led to fights between farmers and cattle men, and various objects such as cutlasses/machetes, arrows and wooden objects are used in inflicting injuries during fight [4,53]. This is unlike European and American studies where most of the fights occurred in the streets, clubs and pubs [6,7,10,11].

Also, the increase in maxillofacial injuries due to sports injuries and industrial accidents could be attributed to increase involvement of Nigerians in recreational and professional sport activities, and increase in the numbers of industries over the years without corresponding increase in protective measures. Onyeaso and Adegbesan [64] in a survey among Nigerian sport persons reported that only one-third of them ever used protective elements during sporting activities, whereas about 60% of them have had one form of orofacial injury or the other before.

Maxillofacial skeletal and soft injuries due to animal attacks were not infrequent, especially in northern part of the country [4,51,52]. While dogs remain the animals most commonly implicated in other reports [65,66], cows, camels and donkeys were mostly involved in Nigeria, because cattle rearing and use of animals as "beasts of burden" are still prevalent practices in northern part of Nigeria [4,51,52].

The peak age of incidence of maxillofacial injuries of 21–30 years among Nigerians is not different from reports from other parts of the world [5-10,12-14,57]. The possible explanation for this is that people in this age group take part in dangerous exercises and sports, drive motor vehicles carelessly, and are most likely to be involved in violence [16].

More males were involved in maxillofacial injuries than females in agreement with previous reports [5-10,13,14]. However, a tendency towards an equal male-to female ratio was observed between earlier and later studies in most centres across the country. This can be attributed to a changing workforce. Women, who are used to stay at home, now work in outdoor and high-risk occupations, thus becoming exposed to RTC and other causes of maxillofacial injuries [18,50].

Most of the fractures of maxillofacial skeleton in Nigerian patients were of the mandible, the findings comparable to other reports [9,12-14]. The mobility of the mandible and the fact that it has less bony support than the maxilla have been implicated [16,67]. Dentoalveolar and condylar fractures were reported to be less in Nigerian patients [1,9,12-14,54]. Dental/dentoalveolar injury is frequently overlooked in surveys that review maxillofacial injury [68-70]. Only the analysis of a large number of injuries reveals the risk of suffering from dentoalveolar trauma [68-70]. Gassner et al [69] in a large series of 9,543 patients with 21,067 maxillofacial injuries reported an incidence of 49.9% of dentoalveolar injuries among their patients. Gassner et al [70] in another large series of craniomaxillofacial trauma in 3,385 children younger than 15 years of age reported an incidence of 76.3% cases of dentoalveolar injuries. Midfacial bone fractures especially LeFort types and orbital floor fractures were reported to be commoner than mandibular fractures [69,70] in contrast to Nigerian reports. A low utilization of technological advances in the imaging of maxillofacial fractures (e.g. CT Scan) in Nigeria may be partially responsible for the observed difference. The midfacial skeleton is much more difficult to assess using plain films than is the mandible [71]. The presence of thin bones, fluid-filled spaces (e.g. congested sinuses), and soft tissues (e.g. orbital contents) make accurate assessment difficult with images that do not offer a high degree of contrast [71]. The difference in the incidence of middle-third fractures has also been related to the refusal of Nigerian motorists to use safety devices, which has reduced their survival after severe middle-third fractures [50].

Although, open reduction and internal fixation remains the "gold standard" of treatment of maxillofacial fractures [72,73], this form of treatment however, has not become popular in our environment [1,50]. Presently, the full compliment of equipment and materials for rigid fixation is not readily available in all parts of the country; and where available, the cost of treatment is usually quite prohibitive [45]. Previous Nigerian reports have, however attested to the satisfactory results obtained using simple conservative methods of closed reduction and mandibulo-maxillary fixation [1,4,16,19,21,25,32,36,45,50,54].

## Conclusion

No apparent shift from road traffic crashes as the leading cause of maxillofacial injuries in Nigeria over a period of 40 years was observed, unlike in most developed countries where assaults/interpersonal violence has replaced road traffic crashes as the major cause of the injuries. Injuries have causes, they do not simply befall us from fate or bad luck. Since no magic pill is envisaged for the prevention of road traffic crashes, we need to take good stock of all the tools at our disposal, and to get down to what the developed nations have done to reduce/prevent road traffic crashes. Therefore, an awareness campaign to educate the public about the importance of restraints and protective headgear in cars and motorcycles should be championed. These findings should also alert the authorities, particularly the government and the Road Safety Commission to the need for the provision of good roads, enforcement of existing traffic laws, and general improvement of socioeconomic condition of the populace.

## Competing interests

The author(s) declare they have no competing interest.

## Authors' contributions

WLA conceived the study and did the literature search, coordinated the write-up and submission of the article. WLA, ALL, MOO and OJ participated in the writing of the manuscript. All the authors read and approved the final manuscript.
